# Nuclear transcription factor Nrf2 suppresses prostate cancer cells growth and migration through upregulating ferroportin

**DOI:** 10.18632/oncotarget.12860

**Published:** 2016-10-24

**Authors:** Dong Xue, Cuixing Zhou, Yunbo Shi, Hao Lu, Renfang Xu, Xiaozhou He

**Affiliations:** ^1^ Department of Urology, Third Affiliated Hospital, Suzhou University, Changzhou 213003, Jiangsu, China; ^2^ Foreign Languages School, Changzhou Institute of Technology, Changzhou 213002, Jiangsu, China

**Keywords:** prostate cancer, ferroportin, Nrf2, ferritin, apoptosis

## Abstract

VTo investigate the effect of nuclear transcription factor Nrf2 on the transcription of Ferroportin (FPN) in prostate cancer cells, and the regulation mechanisms of FPN on cell viability, migration and apoptosis of prostate cancer cells.

Empty vectors, pEGFPC1-Nrf2, pEGFPC1-FPN, Si-FPN and Si-Nrf2 were transfected into prostate cancer cell line PC3. The expression of mRNA and protein were measured by real time-PCR (RT-PCR) and western blot. Cell viability, migration, cycle and apoptosis were tested by CCK-8 assay, wound healing and flow cytometry, respectively. The interaction between FPN and Nrf2 was confirmed by chromatin immunoprecipitation (CHIP) assay.

The viability, migration and mitosis of PC3 cells could be repressed by over-expressed FPN, with decreased intracellular ferritin. The CHIP assay demonstrated that Nrf2 is one transcription factor of FPN and promotes its transcription. With the increase of Nrf2 in PC3 cells, the viability, migration ability and concentration of ferritin were suppressed, while the apoptosis rate was increased. The above effects were counteracted by down-regulating FPN.

FPN could inhibit the prostate cancer cell viability, migration and mitosis, which is also related to a decrease of intracellular ferritin content. In conclusion, Nrf2 suppresses prostate cancer cells viability, migration, and mitosis through upregulating FPN.

## INTRODUCTION

Prostate cancer is the most common cancer in males in western countries, and is one of the leading causes of cancer related deaths [1]. Globally, there are more than 900,000 newly diagnosed prostate cancer cases every year [2].

Previous studies found iron metabolism associated with cancer cell growth and metastasis [3–5]. The accumulation of intracellular irons can promote the growth and aggression of cancer cells, while low levels of intracellular irons can suppress the cell proliferation. It has been reported that a few regulatory factors associated with iron metabolism exhibits relevance to prostate cancer, including Hepcidin [6], redox-sensitive transcription factor (NF-κB) [7] and Ferroportin (FPN) [5]. Among the iron regulators, FPN, a transmembrane protein plays a central role in body iron metabolism by serving as an iron export pump [4].

FPN expressed in the basolateral surface of enterocytes and macrophages of the reticuloendothelial system, is the only known iron exporter in mammalian cells [8, 9]. FPN has been widely studied in cancer research and has proven to be pivotal in the proliferation and metastasis of cancer cells. Pan X et al. demonstrated a remarkably lower expression of FPN in cancer tissues from breast cancer patients compared with normal breast cells [10]. It was also demonstrated in a mouse model that the enhanced expression of FPN after transfection with an expression vector of FPN could inhibit the growth of the cancer cells [11]. Ferritin, as a storage molecule of intracellular irons, shares a reverse tendency with intracellular irons [12]. In a word, the reduction of FPN leads to increased intracellular irons and decreased the iron efflux, which then accelerates cancer growth and metastases. Hepcidin, the ligand of FPN, can regulate intracellular iron efflux by inducing its internalization and degradation [8]. Apart from Hepcidin, FPN gene is also transcriptionally regulated by hypoxia inducible factor-2α (HIF-2α), erythroid 2p45 (NF-E2) -related factor 2 (Nrf2) and metal-responsive transcription factor-1 (MTF-1) [13–16].

Nrf2, a basic-region leucine zipper (bZIP) transcription factor, has previously been proven to mediate key proteins expression by upregulating antioxidant-response element (ARE)-related gene transcription [17, 18]. Previous studies have already addressed the importance of Nrf2 in prostate cancer therapy due to its ability to decrease basal reactive oxygen species and make patients more sensitive to radiation therapy [19]. Recently, studies have focused on its crucial role in iron metabolism. Nrf2 has been previously certified to regulate FPN, increase iron efflux and counteract FPN suppression mediated by LPS in macrophages [17]. The potential role of Nrf2 in upregulating FPN is demonstrated in breast cancer research. Chen Y et al. observed a decline of Nrf2 in breast cancer cells in conjunction with the decreased expression of FPN [20], which suggests that Nrf2 can transactivate the FPN expression in breast cancer cells. However, we still do not have a clear understanding of the link between the Nrf2-FPN signalling and prostate cancer's growth and metastases.

Based on the researches above, we came to the hypothesis that Nrf2 could restrict the cell activities of prostate cancer cells through upregulating FPN, which eventually affects iron metabolism. In order to verify this hypothesis, our present study evaluated and compared the level of FNP and Nrf2 between prostatic and normal cancer cells, and explored their effects on the viability, migration, and mitosis of prostate cancer cells.

## RESULTS

### FPN and Nrf2 expression decreased in PC3 cells

**Figure 1:**
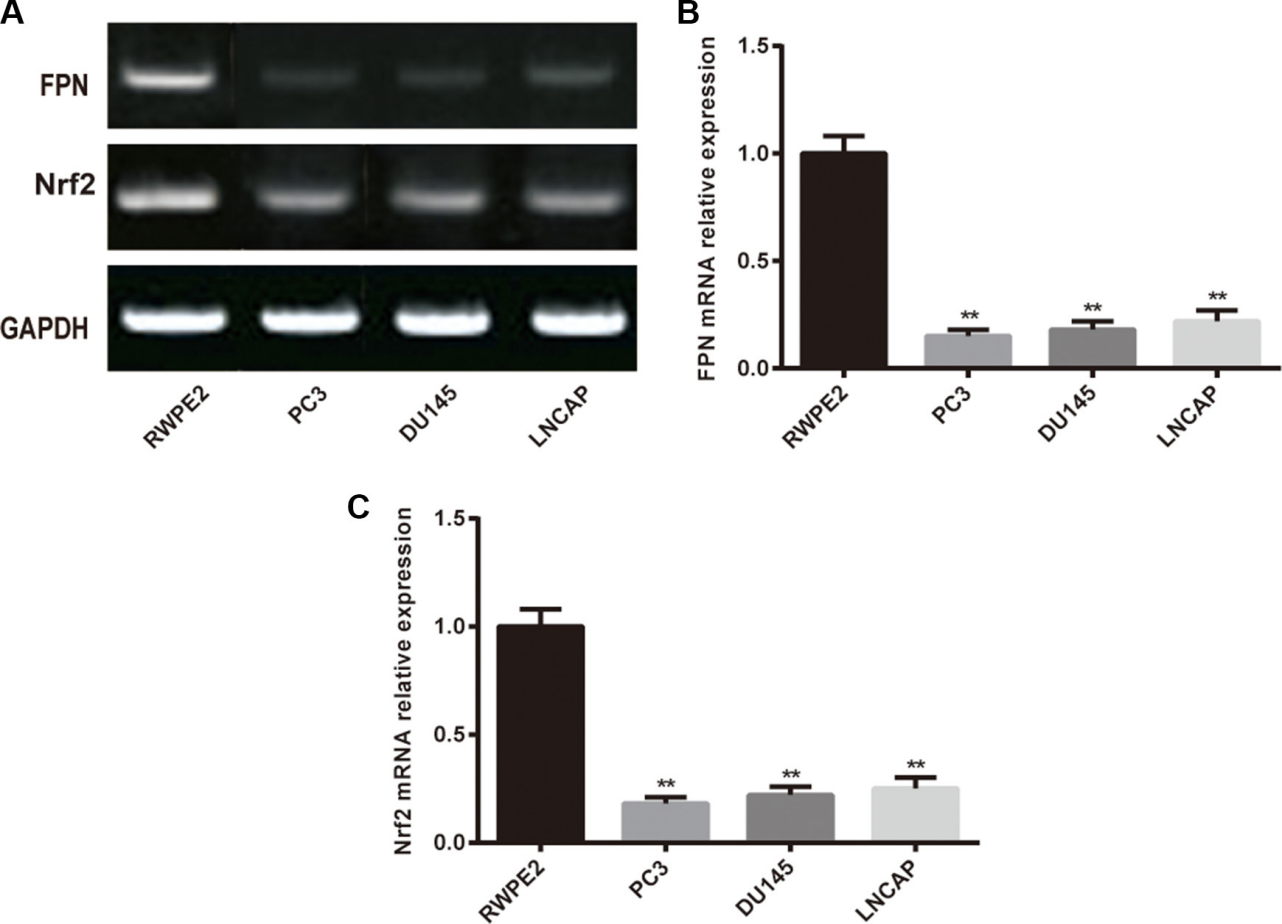
The expression of FPN in RWPE2 cells and prostate cancer cell lines (PC3, DU145 and LNCAP) (**A**) Western blot analysis of FPN and Nrf2 in cells, GAPDH as the internal control. (**B**) RT-PCR analysis mRNA level of FPN in cells. (**C**) RT-PCR analysis mRNA level of Nrf2 in cells. Data are presented as mean ± SD for three independent experiments. ^**^P < 0.01, FPN mRNA expression in PC3 cells versus in RWPE2 cells.

RT-PCR and western blot were used to determine the expression of FPN and Nrf2 in cell lines. As shown in Figure 1, the mRNA expression of FPN and Nrf2 were significantly lower in prostate cancer cell lines (PC3, DU145 and LNCAP) than in RWPE2 cells (*P* < 0.05). Moreover, among the three cancerous cell lines, PC3 showed the most remarkable difference in the expression of FPN and Nrf2 compared with normal prostate cells. Consequently, we chose PC3 for the following assays.

### Nrf2 promoted FPN expression and decreased intracellular ferritin expression

**Figure 2:**
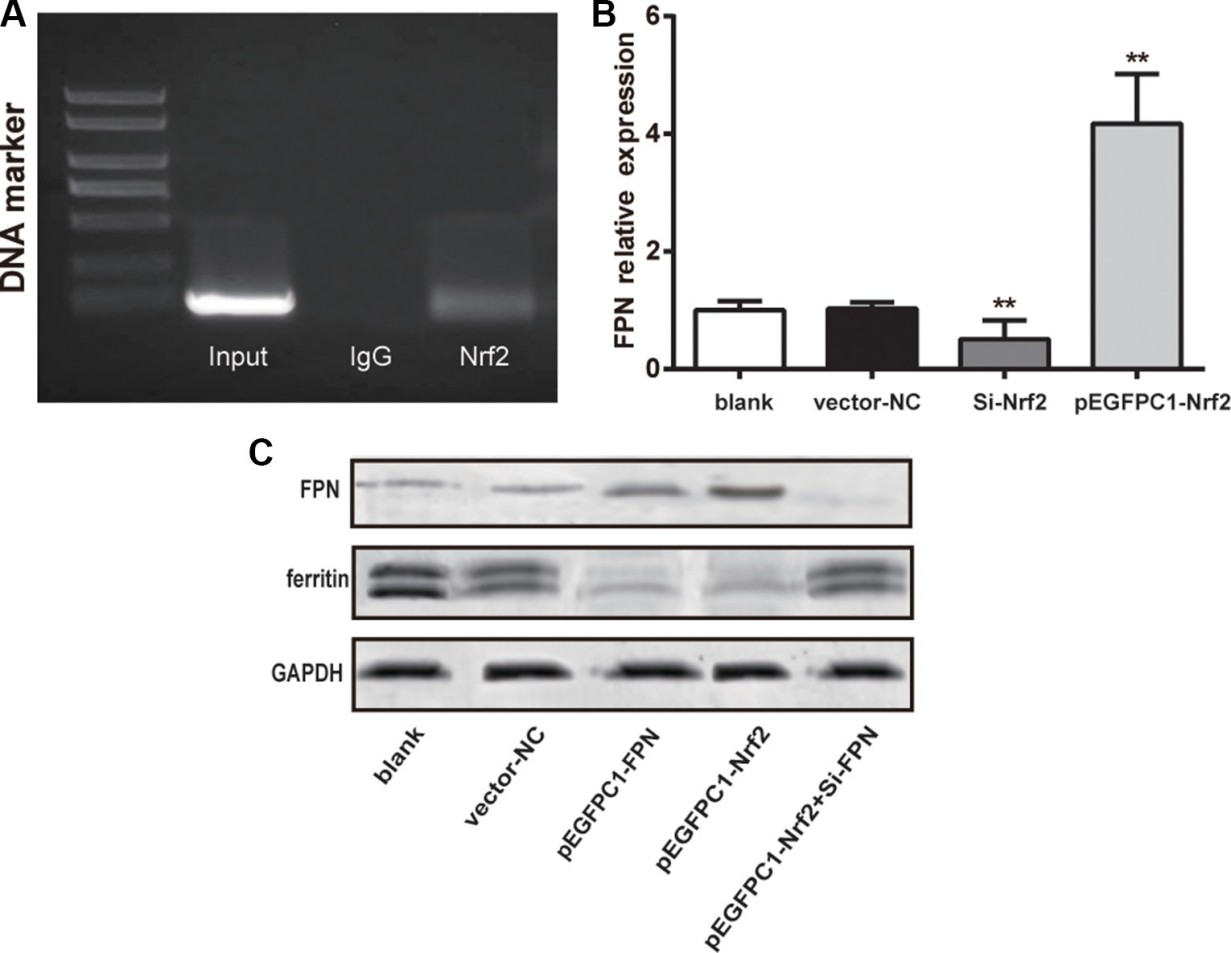
Nrf2 promoted the expression of FPN and decreased ferritin expression (**A**) The PCR results of chromatin immunoprecipitation showing Nrf2 binding to FPN promoter. (**B**) Quantitative data of mRNA level of FPN in different groups. (**C**) Western blot analysis of FPN and ferritin in different five groups, GAPDH as the internal control. Data are presented as mean ± SD for three independent experiments. ^**^P < 0.01 versus the vector–NC group.

The relation between Nrf2 and FPN promoter regions was validated by chromatin immunoprecipitation (CHIP) assay. The electrophoresis result is shown in Figure 2A. The anti-Nrf2 and Input group shared a similar band separation, indicating that Nrf2 could combine with FPN promoter and directly regulated FPN.

Subsequently, we used RT-qPCR to determine the expression of FPN mRNA in the blank, vector-NC, Si-Nrf2 and pEGFPC1-Nrf2 groups. The results are showed in Figure 2B. The Si-Nrf2 group had significant lower FPN mRNA expression compared with the vector–NC group (*P* < 0.01) and the pEGFPC1-Nrf2 group had remarkably higher FPN mRNA expression compared with the vector-NC group (*P* < 0.01). These data demonstrated that Nrf2 was directly involved in FPN expression in prostate cancer cells.

We used western blot analysis to assess the expression of ferritin in PC3 cells in different groups (as shown in Figure 2C). The results show that cells in the pEGFPC1-FPN and pEGFPC1-Nrf2 groups both demonstrated significant lower ferritin expression and higher FPN expression compared with the vector–NC, SiRNA-NC and pEGFPC1-Nrf2+Si-FPN groups. These data demonstrated that upregulated Nrf2 expression improved the expression of FPN, thus led to the decreased intracellular iron contents and increased the iron efflux in PC3 cells.

### FPN influences cell viability, cell cycle, apoptosis and migration in PC3 cells

**Figure 3:**
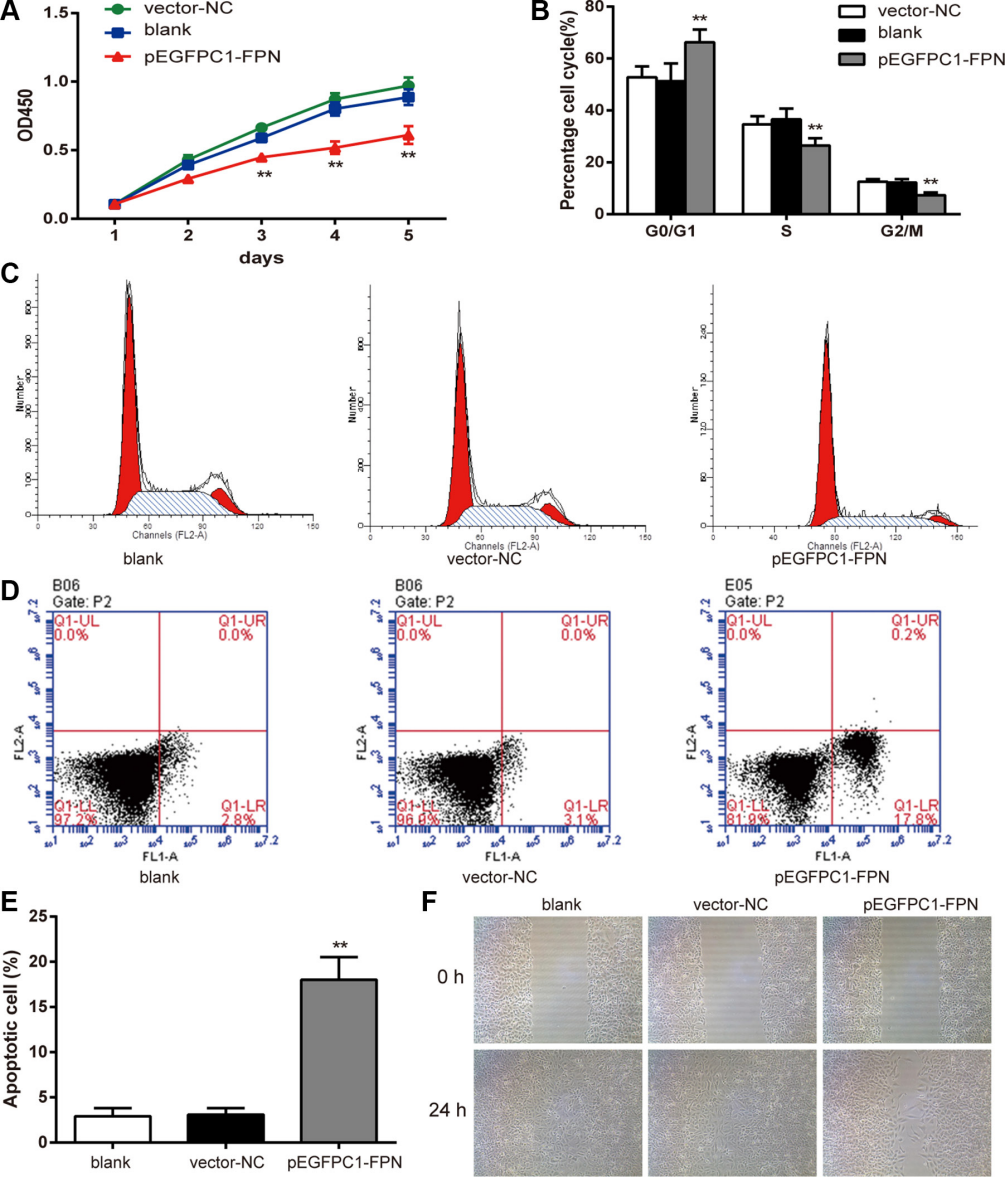
Effects of FPN on the cell viability, cell cycle, apoptosis and migration of PC3 cells (**A**) Effects of FPN on viability of PC3 cells was estimated using CCK-8 assay. (**B**–**C**) Cell cycle was analyzed by flow cytometry. (**D**–**E**) Apoptosis rates of cells in each group were estimated by flow cytometry. (**F**) Effects of FPN on migration of PC3 cells was estimated by wound healing assay. Data are presented as mean ± SD for three independent experiments. ^**^P < 0.01 versus the vector-NC group.

The CCK-8 assay was used to compare the viability of PC3 cells in different groups. The growth curve is shown in Figure 3A. The cell viability of the pEGFPC1-FPN group was significantly lower than that of the vector-NC group (*P* < 0.05). This indicates that the decrease of the viability is related to the high expression of FPN.

Flow cytometry was applied to measure the cell cycle of PC3 cells in different groups. The results are shown in Figure 3B–3C. The ratio of G0/G1 phase cells in the pEGFPC1-FPN group was significant higher than that of the vector-NC group (*P* < 0.05), while the ratio of S phase and G2/M phase cells were significant lower than that of the vector–NC group (*P* < 0.05). The results indicated that upregulated FPN expression inhibited cell cycle transition from G0/G1 phase to S phase and G2/M phase.

In addition, we applied flow cytometry to detect cell apoptosis of the three groups. The results are shown in Figure 3D–3E. The proportion of apoptotic cells in the pEGFPC1-FPN group is significantly higher than that of the vector-NC group (*P* < 0.01), indicating that upregulated FPN expression could promote cell apoptosis in PC3 cells.

Furthermore, to investigate the effects of FPN on PC3 cell migration ability, we examined the cellular migration. In a wound-healing assay, pEGFPC1-FPN group markedly slowed cell migration at the edges of scratch wound of PC3 cells compared to vector-NC group (Figure 3F), indicating that upregulated FPN expression suppresses cellular migration of PC3 cells.

### Nrf2 affects viability, cell cycle, apoptotic and migration of PC3 cells through FPN

**Figure 4:**
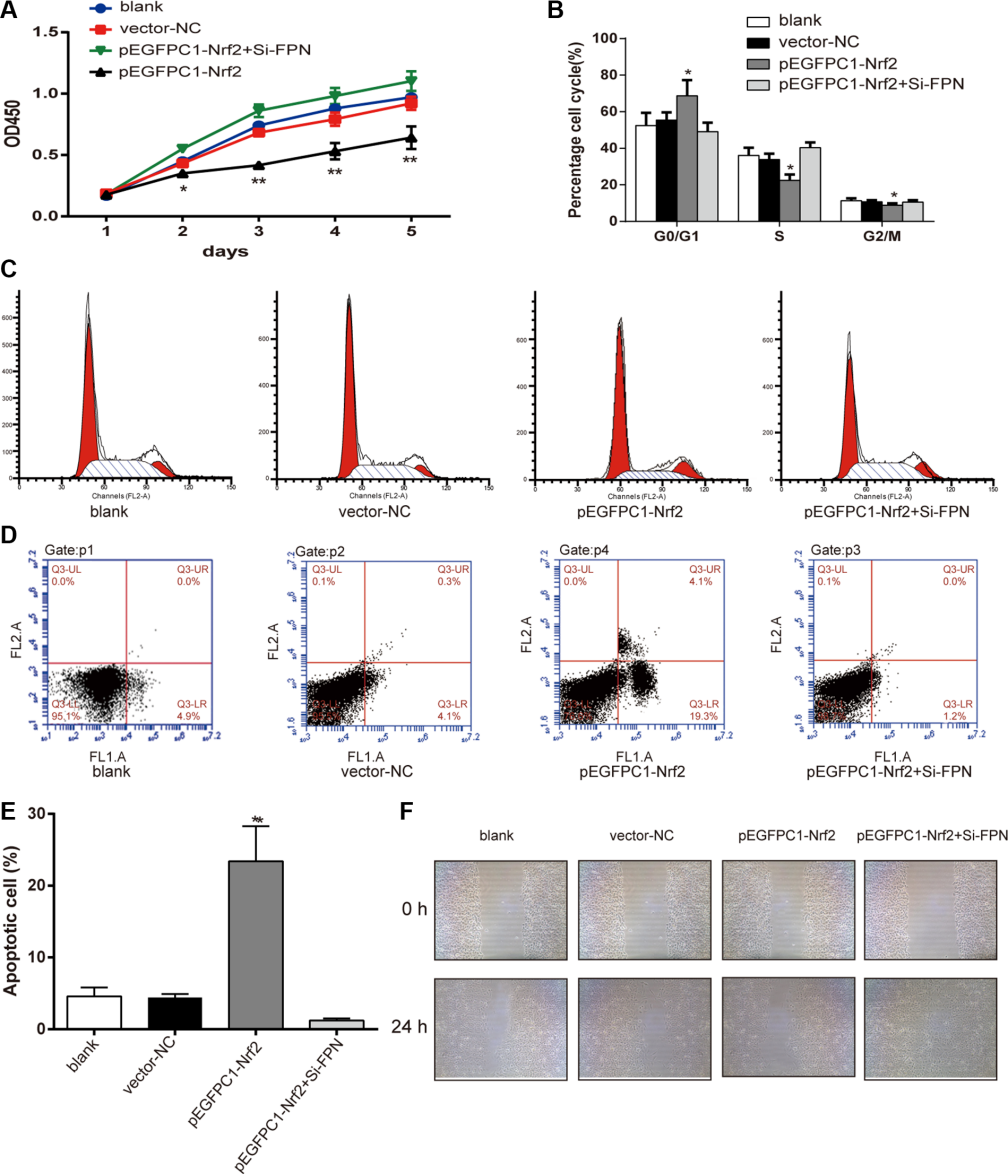
Nrf2 affects the viability, cell cycle, apoptosis and migration of PC3 cells (**A**) Effects of Nrf2 on proliferation of PC3 cells was estimated using CCK-8 assay. (**B**–**C**) Cell cycle was analyzed by flow cytometry. (**D**–**E**) Apoptosis rates of cells in each group were estimated by flow cytometry. (**F**) Effects of Nrf2 on migration of PC3 cells was estimated using wound healing assay. Data are presented as mean ± SD for three independent experiments. ^*^P < 0.05, ^**^P < 0.01 versus vector-NC group, ^#^*P* < 0.05, ^##^*P* < 0.01 versus pEGFPC1-Nrf2+Si-FPN group.

The CCK-8 assay was used to determine the viability of PC3 cells. As shown in Figure 4A, there is no significant difference in cell viability among the blank, vector-NC and pEGFPC1-Nrf2+Si-FPN groups (*P* > 0.05). The viability of cells in the pEGFPC1-Nrf2 group was significantly lower than that of the vector-NC groups (*P* < 0.01). These data demonstrated that upregulated Nrf2 expression suppressed cell viability in PC3 cells, while downregulated FPN expression counteracted the inhibitory effect caused by upregulated Nrf2 expression.

As shown in Figure 4B–4C, the results from the flow cytometry experiment show that there was no difference among blank vector-NC and pEGFPC1-Nrf2+Si-FPN group. However, the ratio of G0/G1 phase cells in the pEGFPC1-Nrf2 group was significantly higher, while the ratio of S phase and G2/M phase cells significantly decreased (*P* < 0.05). These data demonstrated that the upregulation of Nrf2 inhibited cell cycle transition from G0/G1 phase to S phase and G2/M phase compared with vector-NC group, while downregulated FPN expression counteracted the inhibitory effect that caused by upregulated Nrf2 expression. In addition, compared with the vector-NC, SiRNA-NC and pEGFPC1-Nrf2+Si-FPN groups, the pEGFPC1-Nrf2 group had a significantly higher apoptosis rate (*P* < 0.05, Figure 4D–4E).

Furthermore, in the wound-healing assay, cells in pEGFPC1-Nrf2 group demonstrated markedly slower cell migration compared with vector-NC, SiRNA-NC and pEGFPC1-Nrf2+Si-FPN groups (Figure 4F). These data demonstrated that upregulated Nrf2 expression promoted cell apoptosis and suppressed migration of PC3 cells compared with vector-NC cells, while downregulated FPN expression counteracted the promotion or inhibition effect that caused by upregulated Nrf2 expression.

## DISCUSSION

In the introduction part we mentioned the fundamental properties of prostate cancer and the relationship between prostate cancer and iron, the crucial trace mineral that plays a key role in the process of cell growth, angiogenesis and metastasis [21–23]. Particularly, we focused on FPN, the only known iron exporting protein in mammalians [8], which plays an important role in the procedure of body iron transportation by exporting intracellular irons to extracellular environment [4].

Ferritin levels in cells reflect the amount of intracellular irons. Huang et al. proved that the human ferritin, which plays an important role in anti-oxidation, could be down-regulated by the activation of Nrf2-ARE (the antioxidant responsive element) [24]. Moreover, the overexpression of FPN Q248H was reported to reduce the concentration of cellular ferritins [25]. Thus both Nrf2 and FPN could negatively regulate the expression of ferritin, and our results showed a same tendency. When the expression of Nrf2 or FPN was increased in PC3 cells, the concentration of cellular ferritins was suppressed. Then the reduction of cellular ferritins indicated that the intracellular irons were also reduced, which could then inhibit the tumor aggressiveness. Consistently, the ferritin was asserted to directly stimulate the tumorigenesis of breast cancer [26] and the down-regulation of ferritin could suppress the formation of tumors and kill cancer cells [27].

Nrf2 is a crucial transcription factor that is involved in the expression of FPN, and our CHIP assay confirmed that Nrf2 is one of the transcription factors of FPN. Not surprisingly, previous studies also proved that Nrf2 plays a key role in the development of cancers [28–30], including prostate cancer [19, 31]. It seems that an increased expression of Nrf2 may suppress the proliferation and invasion abilities of prostate cancer cells [19, 32], which exactly agreed with the results of our present study. However, Raatikainen et al. revealed that when the Nrf2 was increased, the biochemical recurrence-free survival would be shortened and the overall survival rate would be worse, indicating a worse prognosis of prostate cancer patients and enhanced proliferative and invasive abilities [33]. This seemed to be beyond our expectations that the up-regulation of Nrf2 could suppress cancer development and therefore promised a better survival outcome. Nonetheless, when the Nrf2/FPN signaling lab study turns mature and comes in the clinical trial stage, we would expect the precise regulation of Nrf2/FPN could influence the survival outcome in a good way.

Besides, Xue et al. proved that the expression of FPN was decreased in prostate cancer [4] and we also got a similar result in our research. In addition, our study revealed that the up-regulation of FPN expression led to a decrease in cell proliferation, migration and mitosis of prostate cancer cells, while the down-regulated FPN showed the opposite effects. The results indicated that and FPN was closely associated with the prostate cancer cell development process, which is consistent with the study of Chen et al., who discovered that the FPN expression was reduced in prostate tumors in comparison with adjacent tissues and that FPN could control iron concentration in tumor cells to influence tumor growth [5]. In addition, FPN signaling has been thoroughly studies and it was demonstrated that FPN/Hepcidin signaling involved BMP/SMAD signaling (the iron signaling), JAK/STAT signaling (the inflammatory signaling) and ERFE (the erythropoietic signaling) [34]. Whereas herein we discovered that Nrf2 could be included in the network of the FPN signaling as a novel transcription factor.

Yet the mechanism of the FPN/Nrf2 needs further investigation and we focused on the “targeting” relationship between Nrf2 and FPN, we did contribute to the signaling networking as well as the understanding of the prostate cancer pathogenesis. Besides, we see the future clinical trial a serious challenge as well.

In summary, the current study indicates that the increased Nrf2 levels could stimulate FPN transcriptional expression, leading to the reduction of intracellular ferritins and the suppression of the cell proliferation, migration and mitosis of prostate cancer cells. Our study is a schematic experiment which demonstrated the mechanisms of the Nrf2-mediated regulation of FPN mRNA expression on the prostate tumor cell activities. Besides, this study may provide a new guidance for treating prostate cancer by up-regulating the expression of Nrf2 and we also hope that this study may be helpful for improving the prognosis outcome of prostate cancer patients.

## MATERIALS AND METHODS

### Cell culture

Prostate cancer cell lines PC3, DU145, LNCAP and normal prostate epithelial cell line RWPE2 (purchased from Shanghai Institute for Biological Sciences and Chinese Academy of Sciences) were cultured in Dulbecco modified Eagle medium (DMEM, Gibco, Carlsbad, CA) with 10% fetal bovine serum (FBS, Gibco, Carlsbad, CA) at 37°C under humidified atmosphere with 5% CO_2_.

### Cell transfection

The PCR product of the FPN and Nrf2 was constructed in the vector pEGFPC1. SiRNAs for FPN and Nrf2 were designed and synthesized by Guangzhou RiboBio. Plasmids and SiRNAs transfection were performed using Lipofectamine 2000 according to the manufacturer's instructions (Invitrogen, MA, USA). After 24 hours of incubation, the transfected cells were collected for analysis of mRNA and protein expression. In total, samples were divided into 6 groups: a blank group (cells with no transfection), a vector-NC group (vector negative control, cells transfected with empty vector), a pEGFPC1-FPN group (cells transfected with pEGFPC1-FPN), a Si-Nrf2-group (cells transfected with Nrf2 siRNA), a pEGFPC1-Nrf2 group (cells transfected with pEGFPC1-Nrf2) and a pEGFPC1-Nrf2+Si-FPNgroup (cells transfected with pEGFPC1-Nrf2 and FPN siRNA).

### Real time-PCR (RT-PCR)

The Trizol reagent (Takara, Shiga, Japan) was used to extract the total mRNA from cells. The ReverTra Ace qPCR RT Kit (Fermentas, USA) was used to reversely transcribe total RNA into cDNA. RT-PCR was performed using the THUNDERBIRD SYBR^®^ qPCR Mix (Invitrogen, USA) and CFX96 Touch Real-Time PCR Detection System (Bio-Rad). The relevant primers are listed in Table 1. The PCR for each gene was repeated three times. GAPDH was used as an internal control to normalize FPN and Nrf2 expression. Differential expression of FPN and Nrf2 were calculated using the 2^−ΔΔ*Ct*^ method.

**Table 1:** Primer sequences of GAPDH, FPN, ferritin and Nrf2 for the implementation of RT-PCR

		Primer sequence
GAPDH	Sense	5′-CCACCCAGAAGACTGTGGAT-3′
	Antisense	5′-TTCAGCTCAGGGATGACCTT-3′
FPN	Sense	5′-CGAGATGGATGGGTCTCCTA-3′
	Antisense	5′-ACCACATTTTCGACGTAGCC-3′
Ferritin	Sense	5′-ATGTCCAGCGAAATCCGCCAGA-3′
	Antisense	5′-CAGGAAGAGCAACGTCATCTCA-3′
Nrf2	Sense	5′-CCTAGGCTCCCCTTATCACC-3′
	Antisense	5′-TCACCATGGAGGAAAAGGTC-3′

GAPDH: phosphoglyceraldehyde dehydrogenase, RT-PCR: real time-polymerase chain reaction, FPN: Ferroportin.

### Western-blot

Total cellular proteins were extracted according to the relative kit (Gefan Biological Technology Co., Ltd. Shanghai). Protein concentration was determined with a BCA Protein Assay Kit (Thermo Scientific, America). Proteins were separated by sodium dodecylsulfate–polyacrylamide gel electrophoresis by Bio-rad system, and transferred to the PVDF membranes, the membranes were then blocked with 5% skim milk (Millipore, Billerica, MA, USA). The anti-Nrf2 (Proteintech, Wuhan, China), the anti-FPN (Proteintech Wuhan, China), anti-Ferritin (Proteintech, Wuhan, China) and anti-GAPDH (abcam, USA) (with dilution concentration was 1: 800, 1: 800, 1: 800, 1: 900, respectively) were used as primary antibody, GAPDH as a control reference. Cells were incubated with secondary antibodies for another 2 hours after incubation with primary antibodies at 4°C overnight. The signals were detected using the enhanced chemiluminescence substrate kit (Thermo Fisher Scientific, Rockford, USA), while Image J software was used to analyze results.

### Cell Counting kit-8 (CCK-8) test

Cells (3000–5000/well) were exposed to CCK-8 (Zomanbio, China) after cultured in 96 well-plates for 24 h, 48 h, 72 h, 96 h and 120 h, respectively. After 3 h of incubation with CCK-8, the optical density at 450 nm for each well was read by a spectrophotometer. Experiments were carried out three times.

### Cell migration assay

Wound healing assay was used to test the migration rate of PC3 cells. Briefly, cells were firstly cultured in 6 well-plates with a density of 2 × 10^6^. When cells grew to 80% confluence, a line was scratched vertically in the middle of plates with a pipette tip. After being washed with PBS three times, fresh medium was added, and the wound widths were observed for 24 h. Photographs were taken at 0 h and 24 h using a Nikon Eclipse TS100 microscope (Nikon, Japan).

### Cell cycle and apoptosis assay

For cell cycle assay, 5 × 10^6^ cells in each group were collected and mixed with 70% cold ethanol overnight at 4°C. Cells were then washed with PBS 3 times and then incubated with PBS containing 0.5 mg/ml RNase A and 10 mg/ml propidium iodide for 30 min at 37°C in the dark. The DNA contents distribution was measured with FACS cytometry (BD Biosciences, USA). Each experiment was performed in triplicate.

For cell apoptosis assay, 5 × 10^6^ cells in each group were collected and stained with Annexin V-FITC/PI Apoptosis Detection Kit (BD Biosciences). The apoptosis rates of cells were acquired using FACS cytometry (BD Biosciences, USA). Each experiment was performed in triplicate.

### CHIP assay

Cell samples were incubated with 1% formaldehyde for 10 min at 37°C and subjected to ultrasound to shear DNA into fragments. The CHIP assay was performed using the CHIP EpiQuik Kit (Epigentek). Some chromatins were saved as input control and others diluted in CHIP dilution buffer. The diluted chromatins were incubated with 2μg anti-Nrf2 Ab, or normal immunoglobulin G (IgG). After purification, Immunoprecipitated DNA was analysed using PCR.

### Statistical analysis

All statistical analysis were done using SPSS 18.0 software (Chicago, Illinois, USA). Data were presented in the form of mean ± standard deviation (SD). The two-tailed student's *t*-test was used to analyze the difference between two groups, and one-way variance analysis (ANOVA) together with SNK-q post-test was performed to evaluate the differences among the multiple groups. *P* < 0.05 was equivalent to being statistically significant.

## References

[1] Attard G, Parker C, Eeles RA, Schroder F, Tomlins SA, Tannock I, Drake CG, de Bono JS (2016). Prostate cancer. Lancet.

[2] Dhawan M, Ryan CJ, Ashworth A (2016). DNA Repair Deficiency Is Common in Advanced Prostate Cancer: New Therapeutic Opportunities. Oncologist.

[3] Zhang C, Zhang F (2015). Iron homeostasis and tumorigenesis: molecular mechanisms and therapeutic opportunities. Protein Cell.

[4] Xue D, Zhou CX, Shi YB, Lu H, He XZ (2015). Decreased expression of ferroportin in prostate cancer. Oncol Lett.

[5] Chen Y, Zhang Z, Yang K, Du J, Xu Y, Liu S (2015). Myeloid zinc-finger 1 (MZF-1) suppresses prostate tumor growth through enforcing ferroportin-conducted iron egress. Oncogene.

[6] Nemeth E, Ganz T (2006). Regulation of iron metabolism by hepcidin. Annual review of nutrition.

[7] Ornstein DL, Zacharski LR (2007). Iron stimulates urokinase plasminogen activator expression and activates NF-kappa B in human prostate cancer cells. Nutr Cancer.

[8] Nemeth E, Tuttle MS, Powelson J, Vaughn MB, Donovan A, Ward DM, Ganz T, Kaplan J (2004). Hepcidin regulates cellular iron efflux by binding to ferroportin and inducing its internalization. Science.

[9] Donovan A, Brownlie A, Zhou Y, Shepard J, Pratt SJ, Moynihan J, Paw BH, Drejer A, Barut B, Zapata A, Law TC, Brugnara C, Lux SE (2000). Positional cloning of zebrafish ferroportin1 identifies a conserved vertebrate iron exporter. Nature.

[10] Pan X, Lu Y, Cheng X, Wang J (2016). Hepcidin and ferroportin expression in breast cancer tissue and serum and their relationship with anemia. Curr Oncol.

[11] Pinnix ZK, Miller LD, Wang W, D'Agostino R, Kute T, Willingham MC, Hatcher H, Tesfay L, Sui G, Di X, Torti SV, Torti FM (2010). Ferroportin and iron regulation in breast cancer progression and prognosis. Sci Transl Med.

[12] Cohen B, Dafni H, Meir G, Harmelin A, Neeman M (2005). Ferritin as an endogenous MRI reporter for noninvasive imaging of gene expression in C6 glioma tumors. Neoplasia.

[13] Ward DM, Kaplan J (2012). Ferroportin-mediated iron transport: expression and regulation. Biochim Biophys Acta.

[14] Troadec MB, Ward DM, Lo E, Kaplan J, De Domenico I (2010). Induction of FPN1 transcription by MTF-1 reveals a role for ferroportin in transition metal efflux. Blood.

[15] Taylor M, Qu A, Anderson ER, Matsubara T, Martin A, Gonzalez FJ, Shah YM (2011). Hypoxia-inducible factor-2alpha mediates the adaptive increase of intestinal ferroportin during iron deficiency in mice. Gastroenterology.

[16] Marro S, Chiabrando D, Messana E, Stolte J, Turco E, Tolosano E, Muckenthaler MU (2010). Heme controls ferroportin1 (FPN1) transcription involving Bach1, Nrf2 and a MARE/ARE sequence motif at position -7007 of the FPN1 promoter. Haematologica.

[17] Harada N, Kanayama M, Maruyama A, Yoshida A, Tazumi K, Hosoya T, Mimura J, Toki T, Maher JM, Yamamoto M, Itoh K (2011). Nrf2 regulates ferroportin 1-mediated iron efflux and counteracts lipopolysaccharide-induced ferroportin 1 mRNA suppression in macrophages. Arch Biochem Biophys.

[18] Frohlich DA, McCabe MT, Arnold RS, Day ML (2008). The role of Nrf2 in increased reactive oxygen species and DNA damage in prostate tumorigenesis. Oncogene.

[19] Liu M, Yao XD, Li W, Geng J, Yan Y, Che JP, Xu YF, Zheng JH (2015). Nrf2 sensitizes prostate cancer cells to radiation via decreasing basal ROS levels. BioFactors.

[20] Chen Y, Zhang S, Wang X, Guo W, Wang L, Zhang D, Yuan L, Zhang Z, Xu Y, Liu S (2015). Disordered signaling governing ferroportin transcription favors breast cancer growth. Cell Signal.

[21] Ye L, Kynaston HG, Jiang WG (2007). Bone metastasis in prostate cancer: molecular and cellular mechanisms (Review). Int J Mol Med.

[22] Heath JL, Weiss JM, Lavau CP, Wechsler DS (2013). Iron deprivation in cancer—potential therapeutic implications. Nutrients.

[23] Torti SV, Torti FM (2011). Ironing out cancer. Cancer Res.

[24] Huang BW, Ray PD, Iwasaki K, Tsuji Y (2013). Transcriptional regulation of the human ferritin gene by coordinated regulation of Nrf2 and protein arginine methyltransferases PRMT1 and PRMT4. FASEB journal.

[25] Nekhai S, Xu M, Foster A, Kasvosve I, Diaz S, Machado RF, Castro OL, Kato GJ, Taylor JGt, Gordeuk VR (2013). Reduced sensitivity of the ferroportin Q248H mutant to physiological concentrations of hepcidin. Haematologica.

[26] Alkhateeb AA, Han B, Connor JR (2013). Ferritin stimulates breast cancer cells through an iron-independent mechanism and is localized within tumor-associated macrophages. Breast Cancer Res Treat.

[27] Alkhateeb AA, Connor JR (2013). The significance of ferritin in cancer: anti-oxidation, inflammation and tumorigenesis. Biochim Biophys Acta.

[28] Zhou Y, Li Y, Ni HM, Ding WX, Zhong H (2016). Nrf2 but not autophagy inhibition is associated with the survival of wild-type epidermal growth factor receptor non-small cell lung cancer cells. Toxicol Appl Pharmacol.

[29] Lubelska K, Wiktorska K, Mielczarek L, Milczarek M, Zbroinska-Bregisz I, Chilmonczyk Z (2016). Sulforaphane Regulates NFE2L2/Nrf2-Dependent Xenobiotic Metabolism Phase II, Phase III Enzymes Differently in Human Colorectal Cancer and Untransformed Epithelial Colon Cells. Nutr Cancer.

[30] Kim EH, Jang HJ, Roh JL (2016). A Novel Polyphenol Conjugate Sensitizes Cisplatin-Resistant Head and Neck Cancer Cells to Cisplatin via Nrf2 Inhibition. Mol Cancer Ther.

[31] Schultz MA, Hagan SS, Datta A, Zhang Y, Freeman ML, Sikka SC, Abdel-Mageed AB, Mondal D (2014). Nrf1 and Nrf2 transcription factors regulate androgen receptor transactivation in prostate cancer cells. PloS one.

[32] Li W, Pung D, Su ZY, Guo Y, Zhang C, Yang AY, Zheng X, Du ZY, Zhang K, Kong AN (2016). Epigenetics Reactivation of Nrf2 in Prostate TRAMP C1 Cells by Curcumin Analogue FN1. Chem Res Toxicol.

[33] Raatikainen S, Aaltomaa S, Karja V, Soini Y (2014). Increased nuclear factor erythroid 2-related factor 2 expression predicts worse prognosis of prostate cancer patients treated with radical prostatectomy. Hum Pathol.

[34] Sebastiani G, Wilkinson N, Pantopoulos K (2016). Pharmacological Targeting of the Hepcidin/Ferroportin Axis. Front Pharmacol.

